# M2 macrophages exhibit higher sensitivity to oxLDL-induced lipotoxicity than other monocyte/macrophage subtypes

**DOI:** 10.1186/1476-511X-10-229

**Published:** 2011-12-06

**Authors:** Suleiman A Isa, José S Ruffino, Maninder Ahluwalia, Andrew W Thomas, Keith Morris, Richard Webb

**Affiliations:** 1 Cardiff School of Health Sciences, University of Wales Institute Cardiff, UWIC Llandaf Campus, CARDIFF CF5 2YB, UK

**Keywords:** alternative M2 monocyte/macrophage polarisation, UPR, oxLDL, lipotoxicity

## Abstract

**Background:**

In obesity, phenotypic switches occur in macrophage populations such that the predominantly M2-polarised anti-inflammatory state seen in lean individuals changes to a predominantly M1-polarised pro-inflammatory state in those who are obese. However, the mechanisms by which these phenotypic shifts occur have not yet been fully elucidated.

**Results:**

The effects of oxLDL (1-40 μg/ml; 24 h) on several parameters relevant to the Unfolded Protein Response (UPR)-mediated lipotoxic effects of oxLDL (disruption of ER Ca^2+ ^handling; activation of the UPR transcription factor XBP-1; upregulation of the UPR target genes BiP and CHOP; apoptosis; cell viability) were investigated in human primary monocyte-derived macrophages, and also in monocyte-macrophages derived from the THP-1 monocytic cell line. A consistent pattern was observed: M2-polarised macrophages were more sensitive to the lipotoxic effects of oxLDL than either non-polarised macrophages or non-differentiated monocytic cells. Specifically, M2-polarised macrophages were the only cell type to undergo significantly increased apoptosis (Primary cells: 1.23 ± 0.01 basal; THP-1-derived: 1.97 ± 0.12 basal; *P *< 0.05 in both cases) and decreased cell viability (Primary cells: 0.79 ± 0.04 basal; THP-1-derived: 0.67 ± 0.02 basal; *P *< 0.05 in both cases) when exposed to oxLDL levels similar to those seen in overweight individuals (ie. 1 μg/ml).

**Conclusions:**

We propose that the enhanced susceptibility of M2-polarised macrophages to lipotoxicity seen in the present *in vitro *study could, over time, contribute to the phenotypic shift seen in obese individuals *in vivo*. This is because a higher degree of oxLDL-induced lipotoxic cell death within M2 macrophages could contribute to a decrease in numbers of M2 cells, and thus a relative increase in proportion of non-M2 cells, within macrophage populations. Given the pro-inflammatory characteristics of a predominantly M1-polarised state, the data presented here may constitute a useful contribution to our understanding of the origin of the pro-inflammatory nature of obesity, and of the pathogenesis of obesity-associated inflammatory disorders such as Type 2 Diabetes and atherosclerosis.

## Background

Obesity and associated disorders such as Type-2 Diabetes (T2D) and atherosclerosis are associated with elevated levels of many lipids (eg. increased circulating oxidized low-density lipoprotein (oxLDL) [1,2]), and with chronic inflammation [3]. This can lead to intracellular lipid accumulation in non-adipocyte cells, which can in turn lead to cell death, a phenomenon known as "lipotoxicity" [2].

Due to their wide tissue distribution, monocyte/macrophages, which play vital roles in inflammation and the development of obesity, T2D and atherosclerosis [3], are involved in lipid accumulation within many tissues [4]. For example, the intracellular accumulation of oxLDL within macrophages is mediated by scavenger receptors (such as CD36, SR-A and possibly SR-BI, although the role of the latter is currently controversial [5]) recognizing altered molecular patterns present on oxLDL or other forms of modified lipoproteins such as acetylated LDL (as distinct from non-oxidised LDL), and facilitating its uptake [6].

Two distinct subpopulations of monocyte/macrophages have been identified that exhibit different physiological properties: Th1 cytokines (eg. interferon-γ and interleukin-1β) promote polarisation into proinflammatory "classical" (M1) macrophages, while Th2 cytokines (eg. interleukin-4, interleukin-13) induce polarisation into anti-inflammatory "alternative" (M2) macrophages [7,8]. M2 differentiation is associated with suppressed release of pro-inflammatory mediators [8], enhanced oxidative metabolism [9], and increased mitochondrial biogenesis [10]. Conversely, reductions in M2 differentiation correlate with disruptions in cholesterol homeostasis and reverse cholesterol transport [11], and to increases in total body fat mass, adiposity, glucose intolerance, and insulin resistance [10]. These findings led Odegaard *et al *to suggest that "*macrophage polarisation towards the alternative [M2] state may be a useful strategy for treating T2D*" [10].

Macrophage populations resident in adipose tissue upon high-fat feeding exhibit an M1-predominant state different from that of the M2-predominant population residing in adipose tissue under normal dietary conditions [12]. Moreover, similar phenotypic shifts have been reported for free cholesterol-loaded peritoneal macrophages *in-vitro *[13]. Mechanisms responsible for such obesity-linked shifts to predominantly M1 cells include increased infiltration of M1 cells from the circulation [1], and differentiation of mesenchymal stem cells into M1 cells (or trans-differentation of M2 cells into M1 cells) within adipose tissue [11]. Also, circulating peripheral blood monocytes can be primed for differentiation into functionally distinct macrophage subpopulations in certain circumstances, such as PPARγ-mediated priming of circulating monocytes for differentiation towards an anti-inflammatory M2 macrophage phenotype [8,14]. However, additional mechanism(s) may also underpin the M2-to-M1 shifts seen within pre-existing macrophage populations during the development of obesity [4]; for example, a recent report has suggested that "*lipid-induced toxicity is an important determinant of the obesity-linked proinflammatory switch in macrophage polarisation*" [15].

The endoplasmic reticulum (ER) is a key organelle with regard to lipotoxicity in macrophages, as trafficking of free cholesterol to the ER membrane and incorporation of cholesterol into the normally cholesterol-poor ER membrane alters the physico-chemical properties of this membrane and leads to disruption of ER functions [16]. Disturbance of ER functions leads to the ER becoming overwhelmed with accumulated unfolded proteins, and under such conditions of ER stress, the cell responds by initiating an "unfolded protein response" (UPR [17]). The UPR involves either generalised inhibition of translation combined with specific upregulation of UPR genes that restore the ER's ability to function (eg. binding immunoglobulin protein (BiP; a.k.a. GRP78); [18]), or activation of the pro-apoptotic C/EBP homologous protein (CHOP) pathway [19] if these actions fail to resolve the original ER stress. Hence, UPRs lead either to restoration of normal cell physiology and cell survival, or to cell death [17]. Thus, excessive accumulation of cholesterol into macrophages can lead via UPR/CHOP-triggered apoptosis to lipotoxic macrophage cell death [20,21]; importantly, we and others have recently reported that oxLDL can trigger similar effects [22,23], due to liberation of free cholesterol from oxLDL particles that have been imported into macrophages, and trafficking of this free cholesterol to the ER membrane [16,20,23].

In the present study, we hypothesised that differential sensitivity of M2 macrophages to elevated levels of oxLDL in obesity, and thus to more frequent lipotoxic cell death of M2 cells, may contribute to the decrease in proportion of M2 cells seen within the macrophage population in obese individuals. Thus, using both primary cells and cells derived from the THP-1 monocytic cell lineage, we aimed to evaluate whether oxLDL induced the UPR and lipotoxic apoptosis to the same extent in control and M2-polarised monocyte-derived macrophages.

## Results

### i) Primary Cells

#### IL-13/Rosiglitazone induced upregulation of markers of the M2 macrophage phenotype

**Figure 1:**
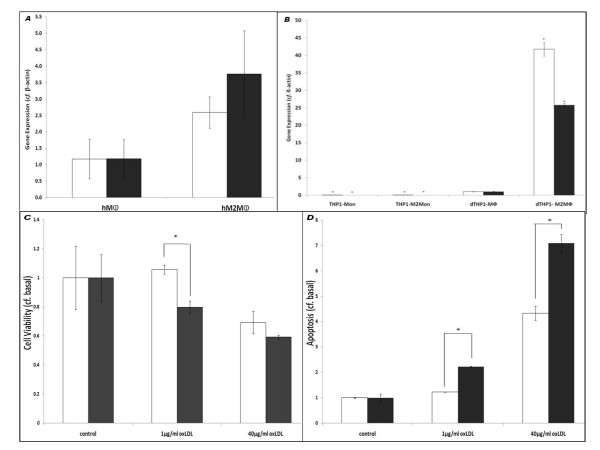
**M2 polarisation and apoptosis/cell viability in primary macrophage cell types**. **A**: RT-PCR was used to determine relative mRNA expression levels for the M2 markers MR (white bars) and IL-Ra (grey bars) before ('hMΦ') and after ('hM2MΦ') treatment of human primary macrophages with 15 ng/mL IL-13/1 μM Rosiglitazone (72 h). n ≥ 3 in all cases; P < 0.10 where indicated. **B**: RT-PCR was used to determine relative mRNA expression levels for the M2 markers MR (white bars) and IL-Ra (grey bars) in THP1-Mon (white bars), THP1-M2Mon (light grey bars), dTHP1-MΦ (dark grey bars) and dTHP1-M2MΦ (black bars) n ≥ 3 in all cases; P < 0.05 where indicated. Spectrophotometric MTS assays (**C**) and luminometric Caspase 3/7 assays (**D**) were used to determine cell viability and apoptosis respectively in hMΦ (white bars) and hM2MΦ (black bars), after treatment with oxLDL (1 μg/ml or 40 μg/ml; 24 h) as indicated. n ≥ 3 in all cases.

Two markers of the M2 phenotype, MR and IL-1Ra [8], underwent significant increases in mRNA expression after treatment of human primary macrophages (hMΦ) with IL-13 and rosiglitazone (see Methods; MR: 2.93 ± 1.03 basal; IL-1Ra: 3.76 ± 1.32 basal; P < 0.10 in both cases; Figure 1a), while MCP-1 (a marker of the M1 phenotype) did not undergo any significant increase (data not shown). Hence, cells that had undergone this treatment were confirmed as exhibiting the M2 macrophage (hM2MΦ) phenotype. IL-13/rosiglitazone treatment did not have any significant effect on cell viability (data not shown).

#### The lipotoxic effects of oxLDL in M2-polarised primary macrophages

Because ER stress-mediated apoptosis depends on activation of Caspases-3 and -7 [24,25], we sought to determine the effect of oxLDL on apoptosis using a luminometric Caspase 3/7 assay. As shown in Figure 1c, hM2MΦ exhibited significantly higher levels of oxLDL-induced apoptosis (1 μg/ml: 2.22 ± 0.06 basal; 40 μg/ml: 7.09 ± 0.36 basal) than hMΦ (1 μg/ml: 1.23 ± 0.01 basal; 40 μg/ml: 4.33 ± 0.28 basal; *P *< 0.05 hMΦ *v*. hM2MΦ for both oxLDL doses). Similarly, as shown in Figure 1d, hM2MΦ exhibited more marked oxLDL-induced decreases in cell viability (1 μg/ml: 0.79 ± 0.04 basal; 40 μg/ml: 0.59 ± 0.02 basal), than hMΦ (1 μg/ml: 1.06 ± 0.03 basal; 40 μg/ml: 0.69 ± 0.07 basal; P < 0.05 hMΦ *v*. hM2MΦ for 1 μg/ml oxLDL; non-significant for high oxLDL dose). Treatment with non-oxidised LDL did not elicit any increases in apoptosis or decreases in cell viability, whereas thapsigargin (100 nM; 24 h), a "classical" inducer of ER stress [15], induced significant increases in apoptosis or decreases in cell viability in both cases (data not shown). Thus, these primary cell data suggest that M2-polarized cells are more sensitive than other forms of macrophage to the specific lipotoxic effects of oxLDL.

### ii) THP-1 derived cells

To elucidate the mechanism underpinning the above effects, cells derived from the cultured monocytic THP-1 cell line [26] were employed as models for primary monocyte-derived macrophages. The cell types used (see Methods section for details) were: THP-1 monocytic cells (THP1-Mon), M2-polarized THP-1 monocytes (THP1-M2Mon), PMA-differentiated THP-1 macrophages (dTHP1-MΦ), and M2-polarized PMA-differentiated THP-1 macrophages (dTHP1-M2MΦ). We have previously reported that intercalation of oxLDL-derived cholesterol into the ER membrane of oxLDL-treated THP1-Mon cells alters the physico-chemical properties of this membrane (as determined by Electron Paramagnetic Resonance spectrometry [23]), and so disrupts ER membrane protein function (as determined by coupled-enzyme SERCA2b Ca^2+^ATPase assays [23]).

#### Expression of marker genes for the M2 macrophage phenotype

As shown in Figure 1b, specific upregulation of two M2 markers, MR and IL-Ra [8], was observed after 72 h treatment of dTHP1-MΦ with IL-13 and rosiglitazone (MR: 41.72 ± 1.92 basal; IL-1Ra: 25.76 ± 0.48 basal; P < 0.05 in both cases). Hence, cells that had undergone this treatment were confirmed as exhibiting an M2 macrophage-like (dTHP1-M2MΦ) phenotype. In contrast, non-differentiated THP-1 monocytic cells expressed both marker genes only at very low levels in either the presence or the absence of IL-13/rosiglitazone treatment (< 0.1 of that seen in dTHP1-MΦ; Figure 1b). Once again, IL-13/rosiglitazone treatment did not have any significant effect on cell viability (data not shown).

#### Measurement of [Ca^2+^]_cyt_

**Figure 2:**
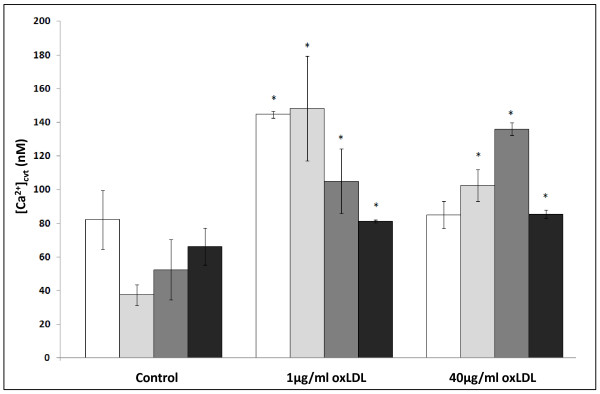
**The effects of oxLDL on cytoplasmic Ca^2+ ^concentrations in THP1-derived monocyte-macrophage cell-types**. Fluo3-mediated fluorimetric quantitation of [Ca^2+^]_cyt _in THP1-Mon (white bars), THP1-M2Mon (light grey bars), dTHP1-MΦ (dark grey bars) and dTHP1-M2MΦ (black bars), after treatment with oxLDL (1 μg/ml or 40 μg/ml; 24 h) as indicated. n ≥ 3 in all cases; P < 0.05 where indicated.

As shown in Figure 2, levels of resting [Ca^2+^]_cyt_, as measured by Fluo-3AM and converted to absolute [Ca^2+^]_cyt _via the Grynkewickz equation [27,28], were within the expected physiological range (i.e. < 0.1 μM; [27]), indicating that this method could reliably be used for the estimation of [Ca^2+^]_cyt_. Under conditions of increased ER membrane rigidity and disrupted ER membrane protein function, Ca^2+ ^can escape from the ER into the cytoplasm [17,29], and in our experiments, treatment with oxLDL (24 h) was associated with significant increases in [Ca^2+^]_cyt _(THP1-Mon: basal: 82 ± 17 nM; 1 μg/ml: 144 ± 30 nM; 40 μg/ml: 85 ± 8 nM; THP1-M2Mon: basal: 38 ± 6 nM; 1 μg/ml: 148 ± 53 nM; 40 μg/ml: 102 ± 16 nM; dTHP1-MΦ: basal: 52 ± 18 nM; 1 μg/ml: 105 ± 33 nM; 40 μg/ml: 135 ± 6 nM; dTHP1-M2MΦ: basal: 66 ± 11 nM; 1 μg/ml: 81 ± 1 nM; 40 μg/ml: 85 ± 4 nM (P < 0.05 v. control in all cases, with the exception of THP1-Mon/40 μg/ml oxLDL, where only a non-significant increase was seen)). 100 nM thapsigargin induced significant increases in [Ca^2+^]_cyt _in all cases (data not shown). Thus, oxLDL appeared to disrupt cellular Ca^2+ ^homeostasis, presumably via its actions on the ER, in all 4 cell types.

#### Oxidized LDL-induced activation of XBP-1

**Figure 3:**
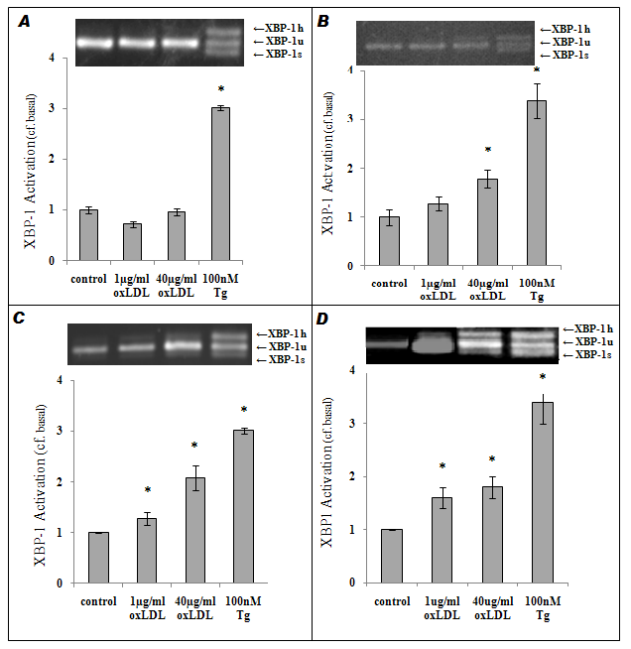
**oxLDL-induced XBP-1 Activation in THP1-derived monocyte-macrophage cell-types**. Representative images (insets) and densitometric summaries from agarose gels used to determine XBP-1 activation in THP1-Mon (**A**), THP1-M2Mon (**B**), dTHP1-MΦ (**C**) and dTHP1-M2MΦ (**D**) after treatment with oxLDL (1 μg/ml or 40 μg/ml; 24 h) or thapsigargin (100 nM; 24 h) as indicated. n ≥ 3 in all cases; P < 0.05 where indicated.

As alternative splicing (and hence activation) of the UPR transcription factor XBP-1 mRNA is an indicator of activation of the UPR in response to ER Stress [17], we employed the methods of Shang *et al *[30] to investigate XBP-1 splicing. Densitometric analysis showed neither 1 μg/ml nor 40 μg/ml oxLDL (24 h in both cases) induced XBP-1 activation in THP1-Mon, while in THP1-M2Mon (Figure 3b) there was XBP-1 activation only at high concentrations of oxLDL (40 μg/ml: 1.79 ± 0.20 basal, P < 0.05). In contrast, both oxLDL doses induced statistically significant XBP-1 activation in dTHP1-MΦ (1 μg/ml: 1.28 ± 0.12 basal; 40 μg/ml: 2.08 ± 0.24 basal; P < 0.05 in both cases; Figure 3c), and dTHP1-M2MΦ cells (1 μg/ml: 1.60 ± 0.20 basal; 40 μg/ml: 1.80 ± 0.20 basal; P < 0.05 in both cases; Figure 3d). As shown in Figure 3, thapsigargin (100 nM; 24 h) induced significantly greater activation in all cases.

#### Determination of mRNA expression levels for the XBP-1 Target Genes BiP and CHOP

**Figure 4:**
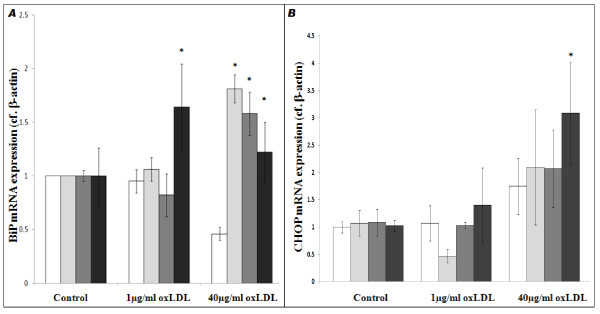
**oxLDL-induced upregulation of the UPR target genes BiP and CHOP in THP1-derived monocyte-macrophage cell-types**. Semi-quantitative determination of BiP (**A**) and CHOP (**B**) mRNA levels in THP1-Mon (white bars), THP1-M2Mon (light grey bars), dTHP1-MΦ (dark grey bars) and dTHP1-M2MΦ (black bars), after treatment with oxLDL (1 μg/ml or 40 μg/ml; 24 h) as indicated. n ≥ 3 in all cases; P < 0.05 where indicated.

To confirm the previous identification of BiP and CHOP as UPR target genes ([18,19]; Figure 4a), we carried out bioinformatics screens of the BiP (Accession No: *NM_005347.3*) and CHOP (Accession No: *NM_001195055.1*) gene sequences. These revealed that within the 5'UTR of the BiP are three ER stress response elements (ERSEs) conforming to the consensus sequence CCAAT(n)_x_CCACA [18] at positions ^-283^, ^-316 ^and ^-348^, while within the CHOP 3'UTR is an ERSE conforming to the same consensus sequence [18] at position ^+661^.

We then investigated the effects exerted by treatment with oxLDL (24 h) on expression of BiP and CHOP; while no oxLDL-induced BiP mRNA induction could be observed in THP1-Mon, oxLDL upregulated BiP at high oxLDL doses in THP1-M2Mon (40 μg/ml: 1.81 ± 0.13 basal (P < 0.05)) and dTHP1-MΦ (40 μg/ml: 1.58 ± 1.20 basal (P < 0.05)), and at both oxLDL doses in dTHP1-M2MΦ (1 μg/ml: 1.64 ± 0.40 basal; 40 μg/ml: 1.44 ± 0.28 basal (P < 0.05 in both cases)). Additionally, as shown in Figure 4b, oxLDL induced only non-significant CHOP upregulation in THP1-Mon, THP1-M2Mon and dTHP1-MΦ. In contrast, in dTHP1-M2MΦ, low oxLDL doses induced non-specific increases in CHOP expression (1.40 ± 0.68 basal; P > 0.05), while high oxLDL doses significantly upregulated CHOP (3.09 ± 0.93 basal; P < 0.05). 100 nM thapsigargin induced significantly greater BiP/CHOP upregulation in all cases (data not shown).

### oxLDL-induced ER stress-mediated apoptosis and decreased cell viability

**Figure 5:**
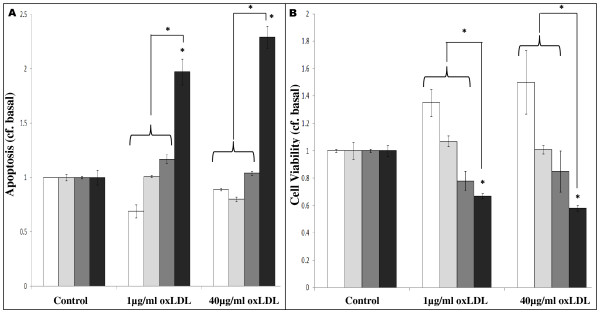
**The effects of oxLDL on apoptosis and cell viability in THP1-derived monocyte-macrophage cell-types**. Luminometric Caspase 3/7 and spectrophotometric MTS assays were used to determine apoptosis (**A**) and cell viability (**B**) respectively in THP1-Mon (white bars), THP1-M2Mon (light grey bars), dTHP1-MΦ (dark grey bars) and dTHP1-M2MΦ (black bars), after treatment with oxLDL (1 μg/ml or 40 μg/ml; 24 h) as indicated. n ≥ 3 in all cases; P < 0.05 where indicated.

Because ER stress-mediated apoptosis depends on activation of Caspases-3 and -7 [24,25], we sought to determine the effect of oxLDL on apoptosis using a luminometric Caspase 3/7 assay (Figure 5a). Treatment with oxLDL did not induce apoptosis in THP1-Mon or THP1-M2Mon, and induced only small non-significant increases in apoptosis in dTHP1-MΦ. However, in dTHP1-M2MΦ, oxLDL induced statistically significant increases in apoptosis (1 μg/ml: 1.97 ± 0.12 basal; 40 μg/ml: 2.29 ± 0.10 basal; P < 0.05 in both cases). Finally, the impact of oxLDL-induced apoptosis on cell viability was investigated: as shown in Figure 5b, no significant decrease in cell viability was observed with either oxLDL dose in either THP1-Mon or THP1-M2Mon. In contrast, both 1 and 40 μg/ml oxLDL induced relatively small decreases in cell viability in dTHP1-MΦ (1 μg/ml: 0.78 ± 0.07-fold, P < 0.05; 40 μg/ml: 0.85 ± 0.15-fold; P > 0.05), while inducing more marked decreases in cell viability in dTHP1-M2MΦ (1 μg/ml: 0.67 ± 0.02-fold; 40 μg/ml: 0.58 ± 0.02-fold; P < 0.05 in both cases; Figure 5b). 100 nM thapsigargin induced significantly greater increased apoptosis or decreased cell viability in all cases (data not shown). As with primary monocyte-derived macrophages, treatment with non-oxidised LDL did not elicit any increases in apoptosis or decreases in cell viability (data not shown).

Importantly, it should be noted that, with regard to apoptosis and cell viability, oxLDL-treated dTHP1-M2MΦ responded to a significantly greater extent than other cell-types (P < 0.05 v. oxLDL-treated THP1-Mon, THP1-M2Mon or dTHP1-MΦ; Figure 5).

## Discussion

The current study presents data indicating that M2-polarised macrophages are more sensitive to the lipotoxic effects of oxLDL than other forms of monocyte/macrophage. The effects of oxLDL on disruption of ER Ca^2+ ^homeostasis, activation of the UPR transcription factor XBP-1, upregulation of the UPR target genes BiP and CHOP, and on apoptosis and cell viability, were determined in six cell types: hMΦ, hM2MΦ, THP1-Mon, THP1-M2Mon, dTHP1-MΦ, and dTHP1-M2MΦ. With regard to these parameters, a consistent pattern was observed: THP1-Mon were the least sensitive to oxLDL, while hM2MΦ and dTHP1-M2MΦ were the most sensitive, with hMΦ, dTHP1-MΦ and THP1-M2Mon being intermediate in their sensitivity. Importantly, the M2-polarised macrophages (hM2MΦ and dTHP1-M2MΦ) were the only cell types to undergo significantly increased apoptosis and decreased cell viability when exposed to oxLDL levels similar to those seen in the circulation of overweight individuals (ie. ~1 μg/ml - as seen in individuals with a body mass index of 25-30 kg/m^2 ^[31]).

The source of oxLDL-induced lipotoxicity in this instance is likely to be cholesterol, a component of oxLDL particles. It has been known for many years that intracellular accumulation of cholesterol results when macrophages take in more lipid than can be excreted; lysosomal cholesterol esterases are responsible for the generation of free cholesterol from lipoprotein-derived cholesteryl esters, and a proportion of the resulting free cholesterol is trafficked to intracellular membranes such as the ER membrane [32]. The ER membrane is a narrow, fluid structure containing < 10% cholesterol [33]; elevation of its cholesterol content results in a broader, less fluid structure which restricts the ability of ER integral membrane proteins to undergo conformational movements and so catalyse their respective enzymatic reactions [22,23]. This results in disrupted ER function [23], and the resulting ER stress has been linked via the UPR/CHOP pathway to the triggering of apoptosis [19,20]. Due to the presence of an ERSE in its 3'UTR (see Results section), CHOP mRNA can be induced by prolonged and/or severe ER stresses to act as a transcription factor that controls several genes associated with apoptosis, including Bcl-2, GADD34, TRB3 and several caspases, including caspases 3 and 7 [19]. (NB. It should be noted that the autophagic pathway, which can lead to non-apoptotic cell death by engulfing, degrading and recycling of cell organelles and proteins [34], is also associated with ER stress [35]; because in the present study, lipotoxic cell death associated with UPRs was assessed solely by determination of CHOP expression and caspase 3/7 activity, the possibility cannot be ruled out that other forms of cell death may also play a role in obesity-related macrophage phenotypic shifts. Nevertheless, in line with several previous papers [19-22], the current study has focused on CHOP-mediated apoptosis as the predominant mode of lipotoxic cell death in macrophages.)

As stated above, several mechanisms that may contribute to the obesity-linked shift to predominantly M1 cells been identified [1,11]. However, additional mechanism(s) may also underpin the M2-to-M1 shifts seen within pre-existing macrophage populations during the development of obesity [4], and as described above, lipotoxicity has recently been proposed as a potential mechanism by which such shifts may occur [15]. In the light of our demonstration that M2 macrophages exhibit higher sensitivity to oxLDL-induced lipotoxicity than non-M2-polarised macrophages, we propose that a higher degree of oxLDL-induced lipotoxic cell death within M2 macrophages may contribute to the decrease in numbers of M2 cells. Over time, a relative increase in proportion of non-M2 cells and hence a predominantly M1 macrophage population would ensue, leading to development of a more pro-inflammatory milieu in the tissues of obese individuals.

Having demonstrated heightened sensitivity to oxLDL-induced lipotoxicity in M2-polarised macrophages, we next considered the possible sources of this heightened sensitivity (ie. we addressed the question: "*At what point in the ER stress/lipotoxicity pathway do M2 cells become more sensitive?*"). There were no significant differences in oxLDL's impact on [Ca^2+^]_cyt _in the different cell types (Figure 2), and dTHP1-M2MΦ did not react to a greater extent than dTHP1-MΦ in terms of XBP-1 activation (Figure 3). However, significant increases in BiP expression (*cf*. THP1-Mon) were only seen in dTHP1-M2MΦ after treatment with 1 μg/ml oxLDL, with only non-significant increases being seen in other cell types (Figure 4a). Moreover, as shown in Figure 4b, statistically significant increases in CHOP expression were seen only in dTHP1-M2MΦ after treatment with 40 μg/ml oxLDL (a similar pattern was seen in the case of 1 μg/ml oxLDL *v*. CHOP, albeit without attaining statistical significance). Finally, with regard to apoptosis and cell viability, the response of dTHP1-M2MΦ to oxLDL was significantly greater than the comparable responses of other THP-1 derived cell-types (Figure 5). As stated above, with regard to primary cells, the same general pattern was observed: hM2MΦ exhibited significantly more apoptosis and less cell viability than hMΦ when exposed to oxLDL levels similar to those seen in sedentary individuals (i.e. 1 μg/ml [31]; Figures 1c and 1d). Thus, it appears that a factor relevant to the UPR (perhaps involving differences in the ability of the UPR to restore normal cell physiology and so avoid the triggering of apoptosis) may be the source of M2-polarised cells' heightened lipotoxic sensitivity. Further elucidation of the source of this differential lipotoxicity may increase our understanding of the causal connections between obesity and inflammatory diseases such as T2D; however, definitive identification of this source is unfortunately beyond the scope of this preliminary study.

As stated above, macrophages are involved in lipid accumulation in a wide variety of tissues, including adipose tissue [12]. Intriguingly, quantitation of lipid-loading in different macrophage subtypes during the progression of obesity in *ob/ob *mice fed a high-fat diet revealed that, while macrophage lipid-loading in general increased, very few lipid-loaded M2 macrophages could be identified, with lipid-loading and adoption of a foam cell-like appearance being characteristic only of M1 macrophages [15]. One may speculate that this could be explained by an inability of M2 macrophages to tolerate large quantities of intracellular lipid, and thus increased M2 cell death in a high-lipid environment. Such an explanation could also account for the high levels of apoptosis and M1-predominant nature of the macrophage foam cell populations resident in the unstable regions surrounding the lipid cores of atherosclerotic plaques [8,21]. Thus, the relevance of differential macrophage lipotoxicity is likely to extend beyond adipose tissue macrophages. For example, the M2-to-M1 shift seen in Kupffer cells during the progression of liver steatosis has recently led Mandal et al to suggest that "*changes in the M1/M2 phenotypic balance can impact on diverse disease conditions*" [36], a statement which lends increased importance to the need to understand the mechanism(s) by which macrophage polarisation can be affected by phenomena such as lipotoxicity.

Clearly, there are several limitations to the current study (eg. the exclusively *in vitro *nature of the experiments; the lack of direct characterisation of differences in cholesterol→ER trafficking in each cell type). In particular, although the current study has specifically investigated the lipotoxic effects of oxLDL (and demonstrated that non-oxidised LDL is not associated with the same effects), it should also be recognised that *in vivo*, macrophages will be exposed to many other forms of lipid. Previous studies investigating different lipids have demonstrated the need to both activate the UPR/CHOP pathway and engage a scavenger receptor if apoptosis is to be triggered [20], suggesting that only lipoproteins that are ligands for scavenger receptors can trigger lipotoxic cell death. However, more recent studies have employed a lipidomic approach and found that a variety of lipid species underwent obesity-associated increases in levels within adipose tissue macrophages, and were associated with shifts towards the M1 phenotype; amongst these lipids were free cholesterol and saturated fatty acids [15]. Interestingly, the relatively rigid inflexible structures of both these lipids are compatible with disruption of ER membrane functions, and thus the triggering of the UPR. Meanwhile, lipids with more flexible structures that would not be expected to disrupt ER membrane properties (eg. polyunsaturated fatty acids, plasmalogens) were not associated with increases in the M1 subtype [15]. Thus, a capacity for triggering ER stress/UPR-linked apoptosis - and possibly a consequent heightened degree of damage to M2-polarised cells, which appear to be more sensitive to lipotoxicity - may underpin the detrimental effects of a variety of harmful lipids (including, but not limited to, oxLDL). Further studies are required to establish whether this is the case.

## Conclusion

Given the elevated levels of circulating oxLDL and other lipids seen in obesity [2] (and hence the increased likelihood of the *in vitro *effects seen here occurring in the tissues of an obese individual *in vivo*), and also the importance of M1 macrophage-mediated inflammation in the pathogenesis of obesity-related diseases such as T2D and atherosclerosis [3], the current study's demonstration that M2-polarised macrophages are more sensitive to the UPR-mediated lipotoxic effects of oxLDL than other forms of monocyte/macrophage may constitute a useful contribution to our understanding of the origin of the pro-inflammatory nature of obesity, and of the pathogenesis of obesity-associated disorders such as T2D and atherosclerosis.

## Materials & methods

### Materials

All reagents were purchased from Sigma-Aldrich (Poole, UK) unless stated otherwise. oxLDL, rosiglitazone, GW9662 and IL-13 were obtained from Autogen Bioclear (Calne, UK), GlaxoSmithKline (Uxbridge, UK) and R&D Systems (Abingdon, UK), respectively. Human primary monocyte-macrophages and human monocytic THP-1 cell lines were obtained from the Welsh Blood Transfusion Service (Llantrisant, UK) and European Collection of Cell Cultures (Salisbury, UK), respectively.

### Generation of primary human monocyte-derived macrophages

Primary human monocyte-derived macrophages were obtained via the method of Iwashima *et al *[37]. Briefly, 10 ml of heparinised blood was diluted 1:1 in RPMI medium, layered over 10 ml of Histopaque-1077 Ficoll-Hypaque and centrifuged at 400 × G for 20 min. The mononuclear cell suspension was carefully removed from the Ficoll-Hypaque interface, and washed four times (500 × G; 10 min) in Phosphate-Buffered Saline (PBS), and mononuclear cells were then incubated (37°C; 5% CO_2_) for 2 h, after which non-adherent cells (representing non-monocytes) were removed by discarding the media. Following adhesion to tissue culture plasticware for 7 days (with medium being changed every 48 h), monocytes were considered to have differentiated to human primary macrophages (hMΦ). M2-polarized macrophages (hM2MΦ) were then obtained via the method of Bouhlel *et al *[8], in which treatment for 72 h with 15 ng/ml IL-13 and 1 μM rosiglitazone induced M2 polarization.

### Maintenance of THP-1 cells in culture

Human THP-1 cells [25] were manipulated in culture in order to generate four distinct cell types: human THP-1 monocytic cells (THP1-Mon), M2-polarized THP-1 monocytes (THP1-M2Mon), PMA-differentiated THP-1 macrophages (dTHP1-MΦ), and M2-polarized PMA-differentiated THP-1 macrophages (dTHP1-M2MΦ). To generate THP1-M2Mon, dTHP1-MΦ, and dTHP1-M2MΦ, the methods of Tjiu *et al *[38] and Bouhlel *et al *[8] were followed, with minor modifications: (a) THP1-M2Mon: THP-1Mon cells were treated for 72 h with 15 ng/ml IL-13 and 1 μM rosiglitazone; (b) dTHP1-MΦ: THP-1Mon cells were treated with 100 ng/ml PMA for 72 h; cells that adopted an adherent macrophage-like phenotype were selected as THP1MΦ; (c) dTHP1-M2MΦ: THP-1Mon cells were pre-incubated with 100 ng/ml PMA for 6 h before addition of 15 ng/ml IL-13/1 μM rosiglitazone for the remaining 66 h of a total incubation time of 72 h.

### Cell treatments

hMΦ, hM2MΦ, THP1-Mon, THP1-M2Mon, dTHP1-MΦ, and dTHP1-M2MΦ were treated for 24 h with 1 μg/ml oxLDL (which approximates to serum oxLDL levels seen in overweight individuals (ie. those with a body mass index of 25-30 kg/m^2^) [31]) or 40 μg/ml oxLDL (a commonly-used supra-physiological dose [39]). In some cases, control samples were treated with non-oxidised LDL, or with thapsigargin (100 nM) as a positive control for disruption of ER homeostasis [16], respectively. Data were expressed for each sample in comparison to the readings obtained in basal samples (ie. cells of the corresponding cell-type that had not been treated with oxLDL).

### Measurement of resting cytoplasmic Ca^2+ ^concentrations ([Ca^2+^]_cyto_)

[Ca^2+^]_cyto _was measured by the method of Elsner [26], in which fluo-3 fluorescence was used as an indicator of resting [Ca^2+^]_cyto_. Adherent cells seeded into the wells of opaque-walled multi-well plates, (dTHP1-MΦ, dTHP1-M2MΦ; approx 5 × 10^3 ^cells per sample), or cell suspensions (THP1-Mon, THP1-M2Mon; 7 × 10^6^/ml in RPMI 1640) were pre-incubated with 1 or 40 μg/ml oxLDL for 24 h at 37°C, and then incubated with 3 μM fluo-3/AM (Molecular Probes, Eugene, OR) for 40 min at 37°C before washing twice with PBS; THP-1Mon and M2Mon samples were resuspended in RPMI 1640 at a final concentration of 1 × 10^6^/ml. Cell samples in multi-well plates were read using a Tecan Infinite^®^200 multimode microplate fluorimeter with a FITC filter (λ_ex _= 490 nm; λ_em _= 520 nm; Tecan, Dorset, UK). Using the Grynkiewicz equation, and previously reported values for Fluo-3's association constant with Ca^2+ ^[27,28], fluorescence readings were then estimated as absolute Ca^2+ ^concentration data.

### RNA extraction, and XBP-1 activation or gene expression RT-PCR assays

In all cases, total RNA from hMΦ, hM2MΦ, THP1-Mon, THP1-M2Mon, dTHP1-MΦ, and dTHP1-M2MΦ (± treatment with oxLDL or thapsigargin for 24 h as described above) were extracted with Trizol^® ^Reagent (Invitrogen, Paisley, UK), and RNA samples were converted to cDNA using an Applied Biosystems^® ^High-Capacity cDNA Reverse transcription Kit (Invitrogen, Paisley, UK), according to the manufacturer's instructions. Activation of the UPR transcription factor XBP-1 was assessed via Shang's method [30], which uses RT-PCR, agarose gel electrophoresis and densitometric analysis to determine XBP-1 activation. Because Ire1's endoribonuclease activity excises a 44 bp segment from within exon 4 of the unspliced XBP-1 mRNA species (XBP-1(u); Accession No: NM_005080.3) under conditions of ER stress, and so generates a spliced mRNA (XBP-1(s); Accession No: NM_001079539.1) encoding the active form of the protein [14], XBP-1 activation was determined via densitometric analysis of banding patterns on 2% agarose gels by means of the following formula "**[XBP-1(s)+0.5 XBP-1(h)]/[XBP-1(s)+XBP-1(h)+XBP-1(u)]**", where XBP-1(s) is a 398 bp PCR product, XBP-1(u) is a 442 bp PCR product, and XBP-1(h) is an additional PCR product representing a heteroduplex XBP-1 cDNA species [30]. In all cases, XBP-1 activation was then expressed relative to that seen in control THP-1Mon cells.

For gene expression assays, expression of genes of interest (see below) was determined using SYBR^® ^Green or Taqman^® ^Gene Expression Assays and an Applied Biosystems 7500 Real-time PCR system (Applied Biosystems, Warrington, UK). In the case of MR, IL-1Ra and CHOP, semi-quantitative (relative to β-actin and/or Glyceraldehyde Phosphate Dehydrogenase (GAPDH)) SYBR^® ^Green assays were carried out using the following primers:

MR: Fwd: 5'-CCATGGACAATGCGCGAGCG-3'

Rev: 5'-CACCTGTGGCCCAAGACACGT-3'

IL-1Ra: Fwd: 5'-GGCCTCCGCAGTCACCTAATCAC-3'

Rev: 5'-GGACAGGCACATCTTCCCTCCAT-3'

CHOP: Fwd: 5'-GCGTCTAGAATGGCAGCTGAGTCATTGCC-3'

Rev: 5'-GCGTCTAGATCATGCTTGGTGCAGATTC-3'

β-actin: Fwd: 5'-TCCTGTGGCATCCACGAA-3'

Rev: 5'-GAAGCATTTGCGGTGGAC-3'

GAPDH: Fwd: 5'-ATGTTCCAGTATGACTCCACTCACG-3'

Rev: 5'-GAAGACACCAGTAGACTCCACGACA-3'

In the case of BiP, mRNA expression was assessed semi-quantitatively (relative to GAPDH) via Taqman^® ^Gene Expression Assays (Applied Biosystems, Warrington, UK) for BiP (Gene Expression Assay Hs99999174_m1) and GAPDH (Gene Expression Assay Hs99999905_m1).

### Cell viability and apoptosis Assays

Cell viability and apoptosis levels within hMΦ, hM2MΦ, THP1-Mon, THP1-M2Mon, dTHP1-MΦ, and dTHP1-M2MΦ samples (± treatment with oxLDL for 24 h as described above) was determined using 3-(4, 5-Dimethylthiazol-2-yl)-2, 5 diphenyltetrazolium bromide (MTS) and Caspase-Glo 3/7 assays (Promega, Southampton, UK), respectively, according to the manufacturers' instructions. The resulting absorbance or luminescence data was read via Dynex plate-reading spectrophotometer or luminometer (Worthing, UK), respectively.

### Statistical analysis

Data are expressed as mean ± standard error of the mean. Statistical significance was determined via ANOVA or 2-sample *t*-tests as appropriate; significance levels were set at *P *< 0.05 except where stated otherwise.

## Competing interests

The authors declare that they have no competing interests.

## Authors' contributions

SAI undertook the major part of the experimental work, and also participated in writing of the original manuscript. JSR carried out the M2 marker RT-PCR experiments, including design/optimisation of the PCR protocol. MA advised on experimental methodologies (particularly PCR-based work), and acted as reviewer of drafts of the manuscript. AWT advised on experimental methodologies (particularly monocyte/macrophage cell culture), and acted as reviewer of drafts of the manuscript. KM advised on experimental methodologies (particularly statistical analysis and M2 polarisation), and acted as reviewer of drafts of the manuscript. RW acted as corresponding author (ie. was responsible for writing and submitting of the manuscript), carried out overall design and co-ordination of the study, and also contributed towards laboratory work, in particular bioinofrmatics and XBP-1 splicing assays. All authors read and approved the final manuscript.
